# Notes on the Use of Bacteriological Methods of Diagnosis in Private Practice.—III

**Published:** 1898-10-01

**Authors:** J. O. Symes

**Affiliations:** Bacteriologist to the Royal Infirmary, Bristol.


### Medical Progress and Hospital Clinics.

### NOTES ON THE USE OF BACTERIOLOGICAL METHODS OF DIAGNOSIS IN PRIVATE PRACTICE.—III.

By J. O. Symes, M.D.Lond., Bacteriologist to the Royal Infirmary, Bristol.

**Examination of Tuberculous Material, Sputum, &c.**—Tubercle bacilli take up the ordinary stains very slowly; for this reason the more powerful stains, such as carbol-fuchsin, are employed, their action being accelerated by heat. Once stained, however, the bacilli resist the decolorising action of acids, and are thus distinguished from other micro-organisms and from tissues which for purposes of contrast may be stained a second time with methylene blue. Whatever previous manipulations the material to be examined may have undergone, the final method of staining the bacilli is practically always the same, whether we be dealing with the scrapings of tissue, sputum, pus, serous fluid, or urine.

A small drop of the material is placed upon a cover-glass, another cover-glass is placed upon this, and the two firmly pressed together, so that when separated only the very thinnest layer remains. This is allowed to dry, and is then fixed by passing the cover through the flame of a spirit lamp. The coverslips are then placed, with the sputum side down, in a watch-glassful of Ziehl-Neelsen's carbol-fuchsin stain—basic fuchsin 1 part, absol. alcohol 10 parts, sol. acid carbol (1-20) 100 parts—which is heated until steam rises. Having been in this stain for ten minutes, they are taken out, washed with water, and then decolorised by dipping for a few seconds in a 20 per cent. solution of nitric acid. If a counter-stain is desired the cover-slips are again washed in water, and stained a second time for two or three minutes in a watery solution of methylene blue. Personally I prefer to use Fraenkel's modification of the process, and to decolorise and counter-stain at the same time by placing the films in the following solution: Distilled water, 50 parts; abs. alcoh., 30 parts; nitric acid, 20 parts; methylene blue in crystals to saturation. Films are allowed to remain in this until all trace of red colour has disappeared, and been replaced with blue.

**Sputum.**—The sputum first coughed up in the morning is the best to examine, as bacilli are generally much more plentiful in this. The small purulent masses or "lintels" should be the portions selected for treatment. Sometimes it is impossible to find bacilli in the sputum in cases where the physical signs would seem to leave no doubt as to the diagnosis; in these cases it is frequently possible to find them by treating the sputum with potash for a few hours, diluting, and then centrifugalising. Another useful method is to add carbolic acid to the sputum so as to make a strength of 1 in 20, shake for a few minutes, and then centrifugalise.

**Purulent and Serous Effusions.**—In serous effusions, it is a matter of considerable difficulty to detect tubercle bacilli. Very rarely can this be done without centrifugalisation, so that in an investigation of this nature it is important to secure in a sterilised vessel as large a quantity of fluid as possible, for transmission to the laboratory. The above remark applies also to tuberculous urine. In disease of the genito-urinary tract, it is seldom possible to obtain specimens of the bacilli unless the centrifuge has been used. In tuberculous ulceration of the intestine, bacilli may be recognised in the fæces, but the difficulties are very great. Pus should be examined when first it is drawn off, for if the abscess has been opened for some days, it is almost impossible to detect the tubercle bacillus in the discharges. Whether their disappearance is due to the admission of air or to the introduction of other organisms does not appear certain.

In sections of tissues obviously tuberculous, it is often impossible by staining methods to demonstrate the presence of bacilli. Recourse must then be made to histological methods, or, if possible, arrangements must be made for the inoculation of animals with the suspected material.

In searching for tubercle bacilli, as large a number of film preparations as possible should be made, and to fully confirm their absence fresh material should be examined on subsequent occasions.

**Diphtheria and other Throat Affections.**—For purposes of examination material is obtained in one of two ways: Membrane that has been coughed up or been detached by forceps is utilised, or cotton wool swabs which have been smeared over the affected parts are used.

When portions of membrane are obtainable it is sometimes possible, by smearing these on coverslips and then staining with Löffler's methylene blue to detect the Klebs-Löffler bacillus, and thus to establish a diagnosis. Failure to find bacilli by this method should not, however, be regarded as proof that the disease is non-diphtheritic. If culture tubes are available the membrane is passed lightly over the surface of the medium, and the tube sealed, packed, and despatched according to the postal regulations already given.

In the absence of culture outfits the membrane, which should be detached and held with sterile forceps, should be placed in a test-tube or wide-mouthed bottle which has previously been sterilised by boiling. On no account should any preservative be placed in the vessel with the membrane, nor should water be added. One is frequently in receipt of membrane which has been transmitted in antiseptic gauze, or lotion, or in glycerine or spirit, or some other medium which renders it useless for purposes of cultivation. Membrane is best transmitted in a dry vessel securely corked.

When membrane cannot be detached, portions of material are obtained by means of swabs. Before these are used the patient should gargle with distilled or boiled water, or, if an infant, the mouth and nose should be cleansed with a douche of the same fluid. In this way the superficial layer of saliva, mucus, and food *débris* is removed, together with some of the organisms which normally are present in the buccal cavities. Any antiseptic application which may recently have been used is also diluted to such an extent as to render it powerless to interfere with the growth of micro-organisms in the culture which is being taken.

The sterilised swab supplied in most of the "culture outfits" consists of a piece of wool twisted round the end of a copper rod, the whole having been sterilised. This is an instrument which can easily be improvised when occasion arises for its use. The swab should be thoroughly rubbed over the tonsils and pharynx or affected part, care being taken that not merely the tongue and soft palate are touched. If tubes of nutrient media are available the swab is now lightly applied to the surface of the culture medium. There is not much fear of inoculating the tube too lightly; the more common mistake is to apply the swab too gently to the fauces and too vigorously to the surface of the serum, a method of procedure which at a later stage makes it difficult to distinguish the colonies of diphtheria bacilli from those of other micro-organisms. The tube having been inoculated is now ready for despatch to the laboratory for incubation and examination. If culture tubes are not available, the swab is placed in test-tube or bottle, which has been sterilised by boiling, which, after being securely sealed and packed, is to be despatched as quickly as possible. Here, again, it is necessary to say that the containing vessel should be dry, and on no account is any preservative to be added.

In laryngeal diphtheria, although no membrane may be visible in the pharynx or fauces, yet a swabbing of the secretions of these parts, avoiding the tonsil, will nearly always lead to the discovery of the bacillus. Diphtheria, it should be remembered, may attack the nose, middle-ear, conjunctiva or excoriations of the skin, and in taking cultures from these parts the method of procedure is the same as has been described above. Although the typical long forms of diphtheria bacilli are easy to recognise, the shorter atypical forms may readily be confounded with the pseudo-diphtheria bacillus, with the xerosis bacillus, and other micro-organisms.

(*To be concluded.*)

